# Dissolved phosphorus loss in agricultural drainage waters under reduced fertilizer application

**DOI:** 10.1002/jeq2.70339

**Published:** 2026-11-14

**Authors:** Samantha G. Sheahan, Robert C. Barr, Shannon Zezula, Pierre‐Andre Jacinthe

**Affiliations:** ^1^ Department of Earth and Environmental Sciences Indiana University Indianapolis Indianapolis Indiana USA; ^2^ USDA Natural Resource Conservation Service (NRCS) Indiana State Office Indianapolis Indiana USA

## Abstract

Reduction in phosphorus (P) fertilizer input could be a viable water quality protection strategy in agricultural landscapes, but supporting field evidence is lacking. A study was conducted (Indiana) to compare dissolved P loss from two adjacent fields receiving P fertilizer at different rates. The fields were under no‐till, corn (*Zea mays* L.)‐soybean (*Glycine max*) rotation, and cover crops after soybean. During the “high‐P phase” of the study (2016–2017), granulated P fertilizer was broadcast applied to both fields at 87.4 kg P ha^−1^ year^−1^ on average. During the “low‐P phase” (2018–2021), granulated P was applied to one field at an average rate of 31.3 kg P ha^−1^ year^−1^, whereas the other received liquid P fertilizer at 13.4 kg P ha^−1^ year^−1^ on average (thus, 65%–84% reduction in P application). Water discharge and P concentration (dissolved reactive P, DRP; total dissolved P, TDP) in surface runoff and subsurface drainage were monitored. No effect of P fertilizer rate on crop yield was found (corn: 15.2 and 15.7 Mg ha^−1^; soybean: 5.4 and 5.3 Mg ha^−1^ during the high‐P and low‐P phases, respectively). Annual P loss averaged 0.68 kg P ha^−1^ as DRP and 2.9 kg P ha^−1^ as TDP during the “high‐P phase,” but declined considerably during the “low‐P phase” (P loss reduction: 25% for DRP; 72% for TDP). A curvilinear trend (*y* = (0.13 × 1.04^x^) + 0.38, *R* ^2^: 0.78, *p* < 0.001] was observed between TDP loss (*y*) and P application rate (*x*), a trend suggesting that substantial TDP loss is more likely at fertilizer application rates > 40 kg P ha^−1^ year^−1^. These results demonstrate that crop yield can be maintained and water quality protected with a P fertilizer input reduction strategy. Future studies should examine the replicability of these results in drained agricultural fields across different climate zones, soil types, and tillage practices.

## Plain Language Summary

Reduction in the rate of phosphorus fertilizer application could be a viable strategy to control P loss and protect water quality in agricultural regions, but experimental evidence is lacking. We conducted a 6‐year (2016–2021) study in Indiana to monitor crop yield and dissolved P loss in drainage waters from two adjacent fields receiving P fertilizer at different application rates, from 87.4 to 13.4 kg P ha‐1 year‐1. Crop yield was not affected by P application rates. However, substantial declines in the amount of P loss were recorded when the fields were switched from high application of granulated P to liquid injection of P fertilizer. These results demonstrate that crop yield can be maintained and water quality protected with a reduction in P fertilizer application. Because the study was conducted at sites under soil health practices (no‐till, cover crop) for > 10 years, questions remain regarding the transferability of these results to fields under conventional tillage.

**Abbreviations**
DRP: dissolved reactive P
FWMC: flow‐weighted mean concentration
PP: particulate P
TDP: total dissolved P

## 1 INTRODUCTION

Phosphorus (P) is a growth‐limiting nutrient in agroecosystems. In intensive agricultural systems, application of P fertilizer becomes necessary to maintain soil fertility and sustain crop productivity. That is especially true of the US Midwest, a region characterized by intensive agriculture and heavy use of P fertilizers, resulting in progressive topsoil P‐enrichment. For example, an analysis of soil P test records in Ohio has reported a doubling in Bray‐P1 over a 20‐year period (Calhoun et al., 2002). Excess P in soil surface is prone to off‐site export, leading to eutrophication of surface water bodies. In addition, P export from agroecosystems represents a financial loss that may undermine farm profitability.

Due to its reactivity, P tends to accumulate in the surface soil layers (<20 cm) of cropland. As a result of that stratification, substantial P loss can occur via surface runoff depending on rainfall, land topography, and surface roughness (Boardman et al., 2019; Dolph et al., 2019). Due to their flat topography and poor drainage characteristics (fine‐textured soils), most agricultural fields in the US Midwest are equipped with subsurface drainage systems, a network of buried pipes, to facilitate the removal of excess water and allow timely farming operations (Fausey et al., 1987). By facilitating quick water removal, subsurface drainage systems also increase the volume of water passing through the soil profile and consequently the amount of nutrient loss (King et al., 2015).

To reduce P loss from cropland, different management practices have been proposed. Because of its minimal soil disturbance, no till (NT) was thought to be a practice that could help mitigate P loss. However, a meta‐analysis has found that NT decreases particulate P (PP) loss from agroecosystems but increases dissolved P loss (Daryanto et al., 2017). Biopores (dead roots and earthworm channels) in NT soil profile could further facilitate the rapid transmission of water and P via subsurface drainage (Dolph et al., 2019; King et al., 2015). Given these observations, it has been suggested that NT should be complemented with additional conservation practices such as cover crops, precision fertilizer application, and fertilizer‐use reduction to harness the soil quality benefits of NT while minimizing its shortcomings in terms of water quality protection (Daryanto et al., 2020). Past studies have shown mixed effects of NT/cover crop combination on P loss, perhaps due to variability in local climate, season of observation, and cover crop species (Carver et al., 2022; Hanrahan et al., 2021). Thus, although it is well documented that conservation practices can significantly reduce PP loss (Carver et al., 2022; Hanrahan et al., 2021; Smith et al., 2017), their impact on dissolved P loss is debatable.

Various aspects of P fertilizer management have been investigated for their impact on P loss, including fertilizer formulation (liquid and granulated), application method (injection, incorporation, and broadcast) (Tarkalson & Mikkelsen, 2004; Smith et al., 2017), timing (fall vs. spring) (Carver et al., 2022; Dolph et al., 2019), and rate (Carver et al., 2022; King et al., 2015). Most of the research on P fertilizer rate in agriculture has historically focused on crop yield, with little attention on water quality impact (Anthony et al., 2013; Ortas & Islam, 2018). An analysis of data from 62 watersheds across the US Midwest has identified fertilizer application rate as the most important factor controlling P transport into waterways (Boardman et al., 2019). Similarly, national assessments of P sources in Canada (Patoine et al., 2012) and Brazil (Withers et al., 2018) have concluded that reduction in P fertilizer rate could be a viable strategy to reverse water quality degradation in agricultural regions. Several plot‐scale studies (Tarkalson & Mikkelsen, 2004; Algoazany et al., 2007) and data syntheses (Christianson et al., 2016; Kamarath & Yuan, 2023) have reported higher P loss with higher P application rates, lending support to the proposed strategy. However, given the scarcity of field‐scale data for its validation, caution should be exercised before making any final determination regarding the suitability of P fertilizer rate reduction as a viable management strategy (Christianson et al., 2016; Kamarath & Yuan, 2023). Further, some of the available data were obtained under conditions (e.g., rainfall simulation, grassland, and coarse‐textured soils) considerably different from the production systems (corn/soybean) and soil types (poorly‐drained) that are most prevalent in the US Midwest. From a modeling exercise focusing on the Maumee River Watershed (Ohio), Guo et al. (2020) concluded that a 62% reduction in P fertilizer application rate could lead to a 29% reduction in dissolved P loss. Given the similarity in eco‐physiological conditions (row‐crop agriculture, flat landscape, poorly‐drained soils, and subsurface drainage) between that watershed and the US Midwest heartland, these results suggest that reduction in P fertilizer input can be a potential strategy to control P loading into the region's water bodies. However, field‐based experimental evidence to validate that projection is lacking. In addition, questions remain about the potential impact of the strategy on crop yield.

An edge‐of‐field study was initiated to address this information gap. Specific research objectives of the study were to (i) quantify differences in dissolved P loss between adjacent and similarly managed agricultural fields receiving P fertilizer at different application rates (high and low) and (ii) assess the interactive effect of P fertilizer application rate, season and cover crops on P loss.

## 2 MATERIALS AND METHODS

### 2.1 Description of the study sites

The study (2016–2021) was conducted in the School Branch watershed, near Brownsburg, Indiana. The study sites (Lat: 39°53′ N, Long: 86°21′ W) included two adjacent agricultural fields under no‐till, corn (*Zea mays* L.)‐soybean (*Glycine max*) rotation, and cover crops (seeded after soybean harvest) (Table 1). The fields, hereafter referred to as East field (area: 5.67 ha) and West field (4.86 ha), were separated by the School Branch creek. The fields have similar topography (<4% slope) and soil types, mostly Brookston (Typic Argiaquolls) and Crosby (Aeric Epiaqualfs) series.

**TABLE 1:** Crops sequence, along with planting and harvest dates, at the field sites during the study period (2015–2021).

Year	Cash crop			Cover crop		
	Species	Planting date	Harvest date	Species	Seeding date	Termination date
2015	Corn	May 6	October 20			
2016	Soybean	May 25	October 14			
				Oilseed rape	October 14, 2016	April 23, 2017
2017	Corn	May 17	October 25			
2018	Soybean	April 30	October 2			
				Barley	October 3, 2018	May 30, 19
2019	Corn	June 3	October 25			
2020	Soybean	April 11	October 21			
				Wheat	October 10, 2020	September 18, 2021

*Note*: Interseason cover crops are shown in the gray‐shaded rows, along with the seeding and termination date of the cover crop.

Both fields were equipped with a subsurface drainage system (10.2 cm diameter plastic drains). For the East field, the drainage system extended approximately 300 m into the field, yielding an estimated drainage area of influence of 1.2 ha. In the West field, the drain extended about 500 m, yielding an estimated subsurface area of influence of 2 ha (Gentry et al., 2000). A berm was constructed near the bottom of each field to divert surface runoff to an H‐flume.

### 2.2 Phosphorus fertilizer management

Both fields were similarly managed except for P fertilizer application rates. During the first 2 years of the study (2016–2017), both fields received broadcast application of granular monoammonium phosphate (MAP) at an average annual rate of 87.4 kg P ha^−1^ (Table 2); that period was thus referred to as the “high‐P phase.” During the subsequent period 2018–2021 or the “low‐P phase” of the study, the East field was switched to injection of liquid P fertilizer (Pro‐Germ, 9‐24‐3) at an average annual rate of 13.4 kg P ha^−1^, whereas the West field received granulated MAP at an average rate of 31.3 kg P ha^−1^ year^−1^, consistent with Tri‐State (IN, OH, and MI) recommendations (Table 2). Thus, P fertilizer application rate during the “low‐P phase” was 65%–84% lower compared to during the “high‐P phase.” In all cases, P fertilizer was applied around the time of cash crop planting in the spring.

**TABLE 2:** Rates of phosphorus fertilizer application and crop yield at the study sites.

Year	Cash crop	East field		West field	
		P input (kg P ha^−1^)	Crop yield (Mg grain ha^−1^)	P input (kg P ha^−1^)	Crop yield (Mg grain ha^−1^)
2015	Corn	111.2	11.9	111.2	12.9
2016	Soybean	58	5.1	69	4.8
2017	Corn	47.7	15.9	47.7	16.0
2018	Soybean	18.6	6.7	35.1	NA
2019	Corn	17.4	17.7	42.1	18.1
2020	Soybean	6.1	5.7	31.2	5.8
2021	Wheat	11.6	10.1	17	NA†

*Note*: Missing yield data due to technical difficulties with harvesting equipment but, according to the farm owner, yield was similar between the two fields.

### 2.3 Main crops and cover crops

Both fields were in a corn‐soybean rotation (Table 1). After soybean harvest (fall of even years), the fields were seeded with a cover crop, primarily consisting of oilseed rape (*Brassica napus* L.) and barley (*Hordeum vulgare* L.). In fall 2020, winter wheat (*Triticum aestivum* L.) was planted as a harvestable cover crop (Table 1). Cover crop was terminated, using herbicide, in the spring before planting the next corn crop. Crop yield was measured by a combine harvester (John Deere S670) and the data provided by the farmer (Table 2).

### 2.4 Collection and handling of water samples

Each field was equipped with appropriate devices to continuously measure water discharge (surface and subsurface) and collect water samples. Surface runoff was routed to an open‐channel H‐flume (08HS; Tracom) fitted with an ultrasonic level sensor (2110; Teledyne) and interfaced with an ISCO autosampler (model 6712; Teledyne) programmed to collect a water sample for every 11.3 × 10^3^ L of runoff passing through the flume. For subsurface drainage, a trapezoidal flume (LGETRAP; Tracom) was installed at the drain outlet, fitted with an ultrasonic sensor (2110; Teledyne Isco), and interfaced with an autosampler programmed to collect a water sample for every 14.1 × 10^3^ L of drainage water. ISCO autosamplers were loaded with bottles (capacity: 24) lined with Teflon bags. Water samples were retrieved within 12 h following a rainstorm, transported to the laboratory, and processed. A subset (100 mL) of each sample was filtered, and a portion (∼15 mL) of the filtrate was stored in a scintillation vial (kept at 4°C in a refrigerator or frozen if sample cannot be analyzed within 48 h) for P analysis. More information regarding field equipment and water sample custody is available elsewhere (Sheahan, 2025).

### 2.5 Dissolved P analysis

Filtrate was analyzed for dissolved reactive P (DRP) concentration using the ammonium molybdate method (EPA 365.3, revised) adapted for an AQ400 nutrient analyzer (SEAL Analytical).

A portion of each filtrate underwent acid persulfate digestion to determine total dissolved P (TDP). An aliquot (5 mL) of each filtrate was pipetted into a Pyrex tube and mixed with concentrated sulfuric acid (100 µL) and 0.5 mL of potassium persulfate (0.4 M). The mixture was autoclaved (120°C, 30 min, 16.5 psi) to convert organic P into inorganic P. Following autoclaving, the mixture was neutralized (100 µL of 25% NaOH added to 0.5 mL of digested mixture) and analyzed for dissolved reactive P using the ammonium molybdate method described above.

### 2.6 Data processing and computation of P loss

Data (water discharge and P concentration) were organized by calendar year and by flow event for P flux calculation. An event was defined as the period (following the onset of rainfall) between the rise and decrease in water discharge to baseline level, taken as a drainage flow rate that was either zero or remained steadily near zero for at least 12 h. For each event, a flow‐weighted mean concentration (FWMC) of P was calculated as follows:

$$\mathrm{FWMC}=\frac{\sum \left(c_{i}t_{i}q_{i}\right)}{\sum \left(t_{i}q_{i}\right)}$$

where *c* is the sample concentration of *i*th sample during an event (ug P L^−1^), *t* is the time window for the *i*th sample (min), and *q* is the water flow (L min^−1^) associated with the *i*th sample.

For each event, P flux (kg P ha^−1^ event^−1^) was calculated using FWMC (mg L^−1^), volume of water outflow during the event (*Q*; *L*), drainage area (A; ha) of the field, and the following equation:

$$\mathrm{Flux}\;=\left(\mathrm{FWMC}\;\times \;Q\right)/A$$

Using the estimated area of influence of each subsurface drainage system, the volume of drainage water during an event was expressed as water depth equivalent (mm) using the following equation and appropriate conversion factors:

$$\mathrm{Water}\;\mathrm{depth}\;\mathrm{equivalent}=\sum \left(Q\right)\;/A$$

To examine the effect of cover crops on P export, the data were disaggregated by seasons: winter (January–March), spring (April–June), summer (July–August) and fall (October–December). Data were also analyzed for fallow periods without and with cover crops (Table 1).

### 2.7 Statistical analysis

Data were separated by field site, year, and season, with flow events as pseudo replicates, to assess the effect of P fertilizer application rate on dissolved P loss. Repeated measures analysis of variance was conducted using PROC GLM available in the SAS 9.4 software (SAS Institute). Linear regression analysis was used to assess the strength of relationships among variables. Statistical significance was determined at *p* < 0.05.

## 3 RESULTS

### 3.1 Phosphorus fertilizer application rate and crop yield

There was no noticeable change in crop yield with the transition from the “high‐P phase” (2015–2017) to the “low‐P phase” (2018–2021) of the study (Table 2). Corn yield (planted in 2015, 2017, and 2019) in the East and West fields was comparable, averaging 15.2 and 15.7 Mg ha^−1^, respectively (Table 2). Likewise, soybean yield (planted in 2016, 2018, and 2020) averaged 5.4 and 5.3 Mg ha^−1^ in the East and West fields. Overall, across fields and P fertilization regimes, average crop yield was 15.42 ± 2.52 Mg ha^−1^ for corn and 5.62 ± 0.73 for soybean. In 2021, winter wheat was grown as a harvestable cover crop and produced 10.1 Mg ha^−1^ of grains (Table 2).

### 3.2 Rainfall and field hydrology

During the study, the wettest year was 2018 (1236 mm), while the driest year was 2020 (838 mm) (Figure 1). The wet year of 2018 was followed by a very wet spring in 2019, causing a 1‐month delay in corn planting (Table 1). That wet spring of 2019 was followed by a summer marked by several dry‐wet cycles and an abnormally dry fall season (only 97 mm of rainfall between September and December of 2019; Figure 1).

**FIGURE 1:**
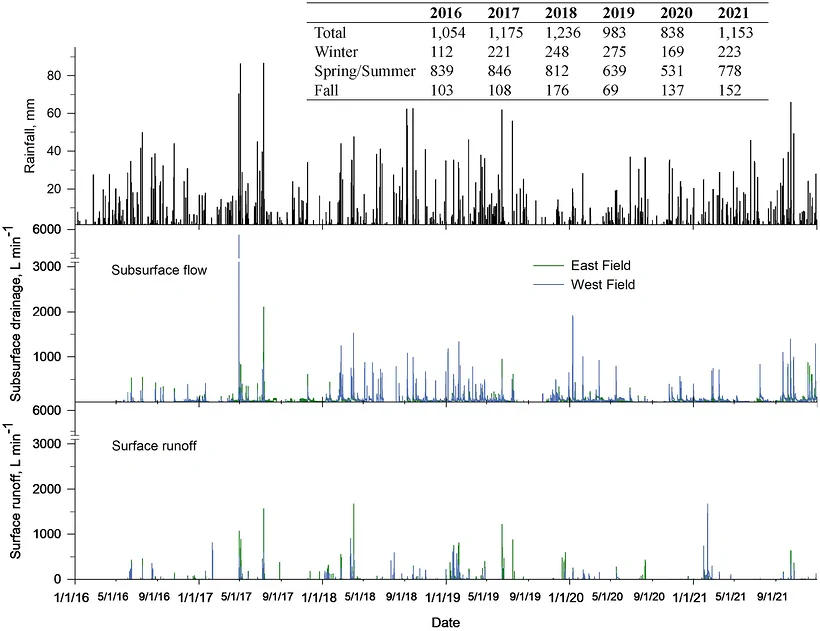
Daily rainfall and drainage water flow rate (subsurface tile and surface runoff) at the study sites during the period 2016–2021. The total amount of rainfall per year and season is reported in the graph insert. Annual normal rainfall is 1108 mm (winter: 235; spring and summer: 712; fall: 162 mm).

Each year, there were on average 27–28 rainstorms that generated subsurface flow (Table 3). Each flow event lasted 3.5 days on average. The frequency of these events varied seasonally and was generally in the order spring > fall > winter > summer (Figure 1; Table 3). The spring season typically accounted for about 30% of all flow events. However, the spring of 2021 noticeably deviated from that trend; no drainage water was collected from either field, although rainfall was near normal (223 mm between March and June compared to a normal of 235 mm) (Figure 1). The fields were planted to winter wheat during that period (Table 1).

**TABLE 3:** Flow‐generating events recorded during the study (2016–2021).

Year	East field				West field			
	Number of events	Cumulative flow volume		Average duration of event	Number of events	Cumulative flow volume		Average duration of event
		(×10^6^ L)	(mm)	(day)		(×10^6^ L)	(mm)	(day)
	Subsurface tile							
2016	16 [0, 4, 4, 8]	1.6	33	2.5	16 [1, 5, 6, 4]	3.35	59.1	3.2
2017	42 [6, 24, 11, 1]	6.88	142	2.6	19 [6, 10, 3, 0]	4.91	86.6	4.0
2018	28 [12, 3, 2, 11]	11.80	243	4.1	35 [10, 8, 2, 15]	15.12	266.7	3.4
2019	31 [9, 14, 2, 6]	12.86	265	5.4	43 [12, 23, 1, 7]	11.11	195.9	2.8
2020	26 [5, 9, 2, 10]	8.43	173	4.5	36 [13, 12, 1, 10]	10.89	192.0	2.5
2021	18 [8, 0, 0, 10]	4.72	97	3.3	19 [7, 0, 1, 11]	7.28	128.5	2.6
	Surface runoff							
2016	3 [0, 0, 2, 1]	0.19	4	0.6	4 [0, 2, 1, 1]	0.42	7	0.5
2017	5 [0, 2, 3, 0]	1.63	34	2.0	6 [1, 3, 1, 0]	0.56	10	0.5
2018	5 [1, 1, 0, 3]	0.83	17	1.1	5 [1, 2, 1, 2]	0.34	6	0.9
2019	7 [2, 3, 2, 0]	2.69	55	1.5	3 [2, 1, 0, 0]	1.24	2	0.3
2020	3 [2, 1, 0, 0]	0.25	5	0.5	3 [2, 1, 0, 0]	0.24	4	0.9
2021	3 [1, 0, 0, 2]	0.38	8	0.3	2 [0, 0, 1, 1]	0.05	1	0.8

*Note*: Values in brackets are the number of events during the winter, spring, summer, and fall seasons. The cumulative amount of drainage water is also expressed as water depth equivalent (mm) using measured drainage area of each field (for surface runoff) or calculated area of influence (for subsurface flow). For the year 2016, the recorded water volume was for a period of 8 months (May—December).

Surface runoff occurred at an average frequency of 4 events per year, mostly when rainfall exceeded 43 mm (Table 3; Figure 1). Surface runoff events lasted 0.8 days on average. When converted to water depth equivalent, subsurface discharge volume corresponded to around 28% of annual rainfall (Table 3), whereas surface runoff only accounted for about 4%. It follows that subsurface drainage accounted for most of the water leaving the fields (Table 3).

### 3.3 Phosphorus concentration in surface runoff and subsurface drainage waters

#### 3.3.1 Dissolved phosphorus concentration in surface runoff

Regardless of transport pathway and P form, both the concentration and flux of P were significantly affected by year of observation. Although limited by the relatively small number of surface runoff events, ANOVA showed a significant effect of sampling year, with P concentration and loss recorded in 2016 being significantly (*p* < 0.001) greater than for the other years (Figure 2).

**FIGURE 2:**
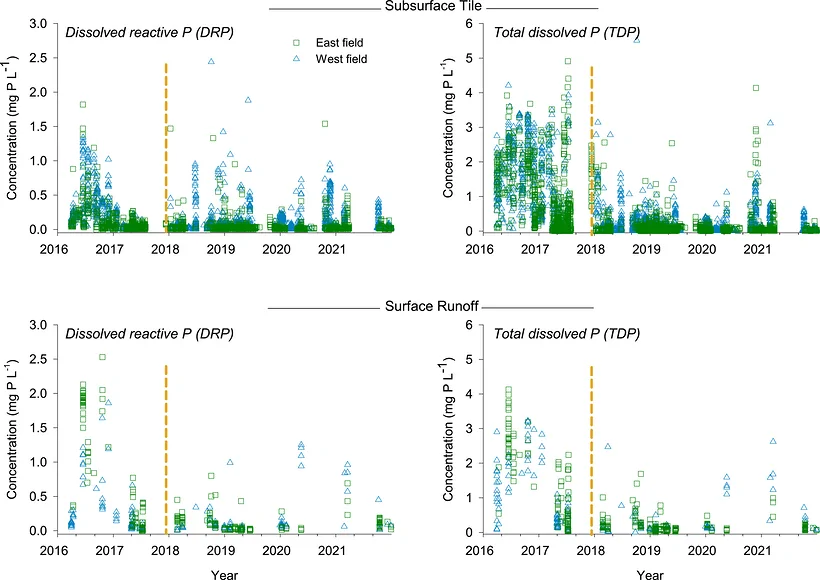
Temporal variation in the concentration of dissolved reactive P (DRP, left panels) and total dissolved P (TDP, right panels) in drainage waters (subsurface tile drainage and surface runoff) during the study (2016–2021). The orange dashed line indicates the transition from the “high‐P input” to “the low‐P input” phases of the study.

The highest concentration of DRP in surface runoff (East: 2.53 and West: 1.86 mg P L^−1^) was recorded during the “high‐P phase” (Figure 2). During that phase, the FWMC of DRP in surface runoff was 0.95 and 0.30 mg P L^−1^ in the East and West fields, respectively. In contrast, during the “low‐P phase” (2018‐2021), FWMC of DRP in surface runoff decreased to 0.10 and 0.24 mg P L^−1^ from these respective fields (Figure 2). In summary, the transition from the “high‐P phase” to the “low‐P phase” resulted in 85% reduction in an DRP concentration in runoff from the East field. The DRP concentration reduction was more modest (37%) in the West field, which continued to receive P fertilizer at recommended Tri‐State rates (Figure 2).

The concentration of TDP in surface runoff followed a similar pattern, with the highest TDP concentration measured in 2016 (East: 4.13; West: 3.22 mg P L^−1^) (Figure 2). A significant (*p* < 0.001) reduction in TDP concentration in surface runoff was also observed during the transition from the “high‐P phase” (East: 1.22 mg P L^−1^ and West: 1.64 mg P L^−1^) to the “low‐P phase” (East: 0.21 mg P L^−1^ and West: 0.52 mg P L^−1^ (Figure 2).

#### 3.3.2 Dissolved phosphorus concentration in subsurface drainage waters

The temporal patterns in DRP concentration in surface runoff and subsurface drainage waters were generally similar. During the “high‐P phase” (2016‐2017), DRP concentration in subsurface waters was 0.16 and 0.18 mg P L^−1^ in the East and West fields, respectively. During the “low‐P phase,” DRP concentration in subsurface drainage waters dropped (>80%) sharply, averaging around 0.02–0.03 mg P L^−1^ (Figure 2).

Similarly, during the “high‐P phase,” TDP concentration in subsurface waters averaged 1.03 and 1.19 mg P L^−1^ for the East and West fields, respectively. During the “low‐P phase,” TDP in drainage waters averaged 0.07 mg P L^−1^ for the East field and 0.11 mg P L^−1^ for the West field (Figure 2). Thus, with implementation of the “low‐P phase,” a substantial reduction (>90% relative to “high‐P phase”) in TDP concentration in subsurface drainage waters was observed.

### 3.4 Annual phosphorus flux via surface runoff and subsurface drainage waters

#### 3.4.1 Phosphorus loss via surface runoff

Across both fields, DRP loss via surface runoff averaged 0.04 kg P ha^−1^ year^−1^ during the “high‐P phase” and was < 0.01 kg P ha^−1^ year^−1^ during the “low‐P phase” (Figure 3). Similarly, TDP loss via surface runoff was low, ranging from 0.03 to 0.14 and from 0.01 to 0.04 kg P ha^−1^ year^−1^ during the “high P” and “low‐P” phases, respectively (Figure 4).

**FIGURE 3:**
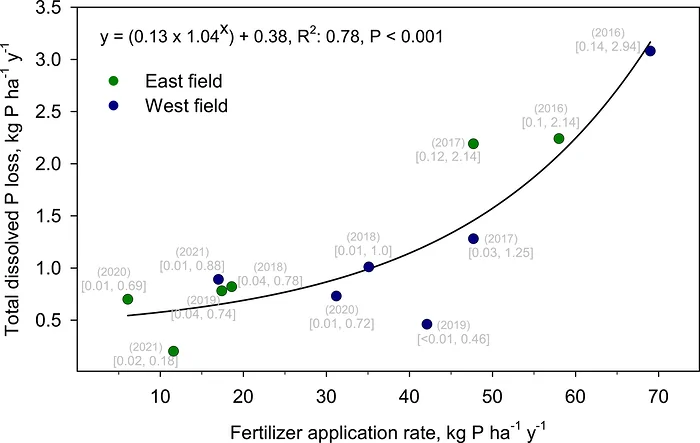
Relationship between phosphorus fertilizer application rate and annual loss of total dissolved P (TDP) loss in drainage waters at study sites. The annotation, next to each data point, indicates the year of monitoring (in parentheses) and the amount of TDP loss via surface runoff (first number in brackets) and subsurface drainage (second number in brackets).

**FIGURE 4:**
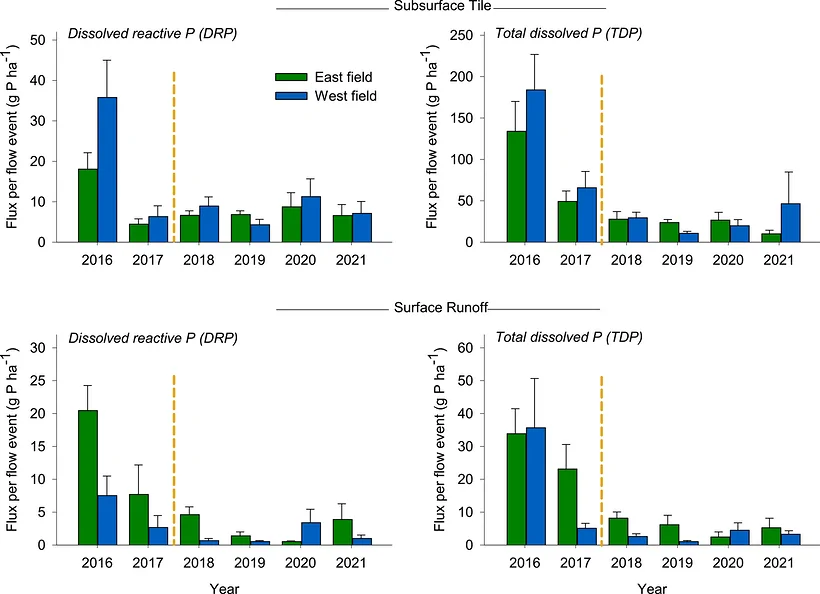
Average rate of P export per hydrologic event as dissolved reactive P (DRP, left panels) and dissolved total P (TDP, right panels) via subsurface tile and surface runoff during the study (2016–2021). The orange dashed line indicates the transition from the “high‐P input” to “the low‐P input” phases of the study.

#### 3.4.2 Phosphorus loss via subsurface drainage

In this study, most (>90%) of the P loss occurred via subsurface drainage (Figure 3). During the “high‐P phase,” DRP export via subsurface drainage averaged 0.24 and 0.29 kg P ha^−1^ year^−1^, respectively, from the East and West fields. During the “low‐P phase,” average DRP loss via subsurface drainage ranged from 0.12 to 0.19 kg P ha^−1^ year^−1^ for the East field and from 0.14 to 0.30 kg P ha^−1^ year^−1^ for the West field (Figure 3). During the “high‐P phase,” TDP loss via subsurface drainage was similar across the two fields, averaging 2.10 kg P ha^−1^ year^−1^. During the “low‐P phase,” TDP loss decreased, averaging 0.60 and 0.76 kg P ha^−1^ year^−1^ at the East and West fields, respectively (Figure 3). A significant (*p* < 0.001) curvilinear relationship (*y* = (0.13 × 1.04^x^) + 0.38, *R* ^2^: 0.78, *p* < 0.001) was found between TDP loss (*y*) and P application rate (*x*).

### 3.5 Seasonal phosphorus loss and effect of cover crops

During the fall season, DRP loss was higher (13.91 g P ha^−1^ event^−1^) when a cover crop was present than when there was no cover crop (3.44 g P ha^−1^ event^−1^) (Figure 5). In contrast, during the winter and spring seasons, DRP loss was consistently lower with cover crops present than without. In the spring, DRP loss was several‐fold lower with cover crops (4.56 g P ha^−1^ event^−1^) than without (16.24 g P ha^−1^ event^−1^) (Figure 5). Similar trends were observed for TDP during the fall and spring seasons.

**FIGURE 5:**
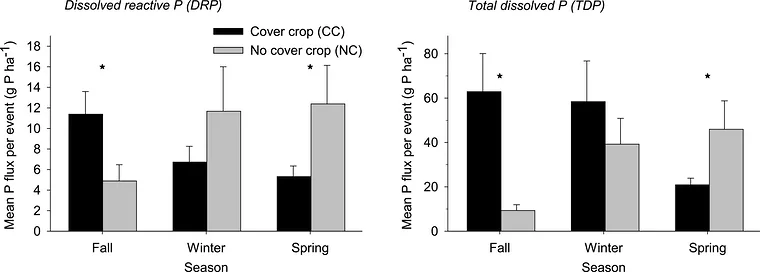
Variation in P export per hydrologic event with cover crop (CC) or without cover crop (NC) during the non‐growing season. For a given season, bars annotated with an asterisk indicate significant difference between CC and NC. The error bars represent standard deviations.

## 4 DISCUSSION

The primary motivation for this study was to determine whether a reduction in P fertilizer application rates can be a viable strategy to control P loss from agroecosystems and protect water quality in agricultural landscapes without incurring a crop yield penalty. Additionally, the study examined the potential impact of cover crops and weather variability on P loss. Results showed that reduction in P fertilizer application rate had no adverse effect on crop yield but resulted in substantial declines in P loss. Further examination of the data revealed a potential effect of dry‐wet cycles on dissolved P loss and a distinct effect of winter wheat, when used as a harvestable cover crop, in curtailing the loss of dissolved P from agroecosystems.

### 4.1 Linkage between phosphorus fertilizer rate and dissolved phosphorus loss

The study was designed to explore the linkage between dissolved P loss in agricultural drainage waters and the rate of P fertilizer application. Indeed, the study results underscore that linkage is exhibited by the curvilinear relationship between TDP loss and P fertilizer application rate. When annual P fertilizer input was < 40 kg ha^−1^, TDP loss measured in this study compared favorably with TDP loss (range: 0.05–1.0 kg ha^−1^ year^−1^) reported for drained fields in Illinois (Gentry et al., 2007) and other agricultural regions (Christianson et al., 2016). However, TDP loss rapidly accelerated when annual P application rate was > 40 kg P ha^−1^, underscoring the environmental impact of excessive P fertilization.

During the “high‐P phase” of the study (2016–2017), when the fields were heavily fertilized (mean application rate: 87.4 kg P ha^−1^ year^−1^), the highest amount of P loss was measured (mean across the two fields: 2.9 kg P ha^−1^ year^−1^) (Figure 3). Conversely, when a reduction (65%–84%) in P fertilizer application was implemented (“low‐P phase”: 2018–2021), there was a noticeable decrease in the loss of both DRP (25%) and TDP (72%) relative to the “high‐P phase.”

It is instructive to note that, during the “low‐P phase,” the year‐to‐year decline in TDP loss was gradual (Figure 4). Although other factors may have contributed, this temporal trend suggests that a decline in TDP loss may not occur immediately with implementation of a low‐P input strategy but, depending on soil types and climate, it may take a few years for the excess legacy‐P to leach out and for the effect of the new P management to become fully noticeable. Prior to this study initiation in 2015, the concentration of water‐extractable P (WEP) in the upper soil layers (0–10 cm) of the East field averaged 12.3 mg P kg^−1^ soil. Immediately following the “high‐P phase,” mean WEP concentration reached 36.5 mg P kg^−1^ in 2017 (Sheahan, 2025). Thus, due to the high rates of P application during the “high‐P phase,” soil P concentration may have approached P saturation level. Therefore, P loss measured during the succeeding “low‐P phase” not only reflected P input during that phase but likely included labile soil P fractions built‐up during the “high‐P phase” of the study. In a study comparing P application methods and P loss via subsurface drainage across study sites in Ohio and Indiana, P loss was found to be related to soil P level (that includes pre‐existing soil P and newly added P), as well as P fertilizer formulation and method of application (Osterholz et al., 2024). In other words, at any given time, the amount of TDP loss in drainage waters reflects both recent P fertilizer application and soil P storage from prior management practices (old P stock or legacy P). The availability of this legacy soil P for transport in drainage waters is controlled by soil chemistry and seasonal rainfall variations, among other factors (Doydora et al., 2020). In the context of the present study, the concentration of WEP nearly tripled after the “high‐P phase” and did not return to pre‐2015 level >3 years under a reduced P fertilization regime (Sheahan, 2025). That temporal trend underscores the long‐term impact of legacy soil P on water quality. Legacy soil P can affect P loss 10–20 years after cessation of conventional practices (e.g., manure, high rate of P fertilizer) and implementation of conservation measures (e.g., the 4R approach, NT, cover cropping) to address water quality concerns (Sharpley, 2016). For many years prior to initiation of the present study, the farmer managing the two fields had already implemented a low‐P management strategy. Therefore, it would have been preferable, during the present study, to first run the “low‐P phase” before the “high‐P phase.”

### 4.2 Impact of seasonal weather variations on phosphorus loss

As has been reported in several past studies (Doydora et al., 2020; Smith & Jacinthe, 2014; Venterink et al., 2002), dry‐wet cycles may have impacted dissolved P loss during the growing season of 2019. Following the 2018 wet year (Figure 1), rainfall distribution in 2019 was characterized by an unusually wet spring (30% above normal rainfall) and then a succession of dry stretches (July rainfall: 24% below normal) interspersed by heavy rainstorms during the fall season (Figure 1). Higher than average concentrations of DRP (0.07 mg P L^−1^) and TDP (0.26 mg P L^−1^) in subsurface drainage waters were recorded on November 26, 2019, following a dry period in September‐October 2019 (79%–86% below normal rainfall) (Figure 2). Likewise, the highest concentrations of DRP (2.4 mg P L^−1^) and TDP (5.5 mg P L^−1^) in drainage waters were recorded on October 5, 2018, when a dry period ended after a large (49.8 mm) rainstorm on October 4, 2018 (Figures 1 and 2). These dry‐wet cycles may have triggered the release of soluble soil P and facilitated its transport to the subsurface drainage system. Various factors have been reported as potential contributors to increased soil P solubilization during dry‐wet cycles, including changes in soil microbial activity (Doydora et al., 2020), the release of osmolytes accumulated in soil microbial cells under dry soil conditions (Schimel et al., 2007), and shift in the control of P activity in soil solution from insoluble to relatively soluble phosphate minerals (Smith & Jacinthe, 2014). Substantial increase in extractable P in wetland soils subjected to extended drying has also been reported (Venterink et al., 2002). Another study (Pitcock & Jacinthe, 2025), using drainage water from this study site, has shown significant increase in enzymatically hydrolyzable P during dry/wet cycles, further highlighting the potential impact of these dry‐wet events on P transformation and loss.

### 4.3 Seasonality of cover crop impact

Cover crops can affect P loss through subsurface drainage waters via evapotranspiration and P uptake. The volume of drainage waters measured during the study (30%–33% of annual rainfall; Table 3) was consistent with several US Midwest studies (31% in Ohio: Pease et al., 2018; 8%–26% in Indiana: Kladivko et al., 2004; 18%–40% in Iowa: Jaynes et al., 2001). Most notably, and consistent with a previous study in Indiana (Trentman et al., 2020), the results showed that subsurface drainage water volume was lower when cover crops were present compared to no cover crops (Figure 1).

The impact of cover crops on dissolved P loss has been investigated across different hydro‐climatic regions and management practices, but reported effects have been inconsistent (Carver et al., 2022; Hanrahan et al., 2021; Smith et al., 2017). Results of this study indicated that, when cover crops were present, DRP loss via subsurface drainage generally increased during the fall season but decreased the following spring season (Figure 5). Except for the high variability observed in the winter, a similar seasonal pattern was observed for TDP loss (fall vs. spring). However, these seasonal patterns should be interpreted with some caution because, in this study, the fields were seeded with cover crops only after soybean harvest (Table 1). It is possible that the increase in P loss observed during the fall season with cover crops was not related to the cover crops per se but perhaps was a consequence of the rapid decomposition of soybean residue during the fall (da Silva et al., 2022). In contrast, during the corn years, the fields were covered with corn residue after fall harvest; since corn residue decomposes more slowly (cereal vs. legume), the amount of P release from crop residue decomposition would consequently be lower. Therefore, differences in crop residue decomposition are an important variable to consider when interpreting P loss in the absence or presence of cover crops.

The most pronounced effect of cover crops on P export was observed when winter wheat was planted as a harvestable cover crop in fall 2020 and remained on the fields for most of the 2021 season. The average rate of DRP loss was in the range of 8.21–18.42 g P ha^−1^ event^−1^ in fall 2020 but was almost nil in spring 2021 despite nearly normal rainfall (Table 3; Figure 4). This is likely a consequence of efficient P scavenging and high rates of evapotranspiration by the winter wheat when it entered its rapid growth phase (extended canopy and root systems; Baker & Gallagher, 1983) during the spring of 2021. Given the reported rates of evapotranspiration (288–435 mm) for this plant (Chen et al., 2010), it is conceivable that most of the available soil water was lost through evapotranspiration instead of entering the subsurface drainage system.

While studies have consistently found a reduction in N loss with cover crops, their impact on P loss has been variable. Similar to the present investigation, another study in Indiana has also reported lower DRP loss during the spring season when cover crops are present (Trentman et al., 2020). However, other studies have reported variable effects of cover crops on P loss in the region (Carver et al., 2022; Hanrahan et al., 2021; Trentman et al., 2020). These variable results may arise from differences in soil fertility, P fertilizer management, tillage, and land‐use history. Results of the present study additionally suggest that the plant species grown as a cover crop is another factor to consider when examining the seasonal impact of cover crops on P loss from agroecosystems. Specifically, these results suggest that winter wheat, due to its growth patterns and phenological traits, could effectively limit P export from cropland to surface water bodies.

## 5 CONCLUSIONS

Fertilizer application rate is a key component of the “4 R” nutrient management framework. In alignment with that concept, a reduction in P fertilizer applied to farmland has been proposed as a strategy to address water quality challenges. The primary motivation for this 6‐year study was to evaluate the viability of the strategy in terms of its impact on dissolved P loss and crop performance.

Results showed that P application rates had no effect on crop yield. A curvilinear relationship was observed between P loss and P application rate, a trend suggesting that acceleration in dissolved P loss is more likely when P application rate exceeds 40 kg P ha^−1^ year^−1^. Overall, the results indicate that the right balance between crop production and water quality protection is achievable and that P loss from agroecosystems can be curtailed through adoption of a P‐input reduction strategy with no adverse effect on crop yield. However, questions remain regarding the impact of that strategy on soil P fertility level in the long term. Further, it should be noted that this study was conducted at sites where soil health practices (no‐till and cover crops) have been implemented for > 10 years, raising questions about the transferability of these results to fields under conventional tillage. Additional investigations, conducted across climate zones and soil types, are needed to validate the results of this study.

The impact of cover crops on dissolved P loss was also examined and found to be variable and dependent on cover crop species, timing of cover crop development, and local weather conditions. Specifically, when a cover crop was present, P loss during the winter and spring seasons was consistently lower than when a cover crop was absent. Winter wheat, planted as a harvestable cover crop in fall 2020, had a noticeable impact on P loss. Despite abundant rainfall, there was hardly any drainage water or P loss in spring 2021. These results underscore the distinct capacity of winter wheat in curtailing dissolved P loss, possibly via increased evapotranspiration and P uptake. The impact of different cover crop species on P loss is not fully understood and warrants further investigations.

## AUTHOR CONTRIBUTIONS

**Samantha G. Sheahan**: Conceptualization; data curation; formal analysis; investigation; methodology; writing—original draft. **Robert C. Barr**: Conceptualization; funding acquisition; resources; writing—review and editing. **Shannon Zezula**: Conceptualization; funding acquisition; resources; writing—review and editing. **Pierre‐Andre Jacinthe**: Conceptualization; funding acquisition; investigation; project administration; writing—review and editing.

## CONFLICT OF INTEREST STATEMENT

The authors declare no conflicts of interest.

## References

[jeq270339-bib-0001] Algoazany, A. S. , Kalita, P. K. , Czapar, G. F. , & Mitchell, J. K. (2007). Phosphorus transport through subsurface drainage and surface runoff from a flat watershed in East Central Illinois, USA. Journal of Environmental Quality, 36, 681–693. 10.2134/jeq2006.0161 17412904

[jeq270339-bib-0002] Anthony, P. M. , Malzer, G. L. , Sparrow, S. D. , & Zhang, M. (2013). Corn and soybean grain phosphorus content relationship with soil phosphorus, phosphorus fertilizer, and crop yield. Communications in Soil Science and Plant Analysis, 44, 1056–1071.

[jeq270339-bib-0003] Baker, C. K. , & Gallagher, J. N. (1983). The development of winter wheat in the field. 1. Relation between apical development and plant morphology within and between seasons. The Journal of Agricultural Science, 101, 327–335.

[jeq270339-bib-0004] Boardman, E. , Danesh‐Yazdi, M. , Foufoula‐Georgiou, E. , Dolph, C. L. , & Finlay, J. C. (2019). Fertilizer, landscape features and climate regulate phosphorus retention and river export in diverse Midwestern watersheds. Biogeochemistry, 146, 293–309.

[jeq270339-bib-0005] Calhoun, F. G. , Bigham, J. M. , & Slater, B. K. (2002). Relationships among plant available phosphorus, fertilizer sales, and water quality in northwestern Ohio. Journal of Environmental Quality, 31, 38–46. 10.2134/jeq2002.3800 11837442

[jeq270339-bib-0006] Carver, R. E. , Nelson, N. O. , Roozeboom, K. L. , Kluitenberg, G. J. , Tomlinson, P. J. , Kang, Q. , & Abel, D. S. (2022). Cover crop and phosphorus fertilizer management impacts on surface water quality from a no‐till corn‐soybean rotation. Journal of Environmental Management, 301, 113818. 10.1016/j.jenvman.2021.113818 34597948

[jeq270339-bib-0007] Chen, S. , Zhang, X. , Sun, H. , Ren, T. , & Wang, Y. (2010). Effects of winter wheat row spacing on evapotranspiration, grain yield and water use efficiency. Agricultural Water Management, 97, 1126–1132.

[jeq270339-bib-0008] Christianson, L. E. , Harmel, R. D. , Smith, D. , Williams, M. R. , & King, K. (2016). Assessment and synthesis of 50 years of published drainage phosphorus losses. Journal of Environmental Quality, 45, 1467–1477. 10.2134/jeq2015.12.0593 27695749

[jeq270339-bib-0009] Daryanto, S. , Wang, L. , & Jacinthe, P. A. (2017). Meta‐analysis of phosphorus loss from no‐till soils. Journal of Environmental Quality, 46, 1028–1037. 10.2134/jeq2017.03.0121 28991965

[jeq270339-bib-0010] Daryanto, S. , Wang, L. , & Jacinthe, P. A. (2020). No‐till is challenged: Complementary management is crucial to improve its environmental benefits under a changing climate. Geography and Sustainability, 1, 229–232.

[jeq270339-bib-0011] da Silva, J. P. , Teixeira, R. D. S. , da Silva, I. R. , Soares, E. M. , & Lima, A. M. (2022). Decomposition and nutrient release from legume and non‐legume residues in a tropical soil. European Journal of Soil Science, 73, e13151.

[jeq270339-bib-0012] Dolph, C. L. , Boardman, E. , Danesh‐Yazdi, M. , Finlay, J. C. , Hansen, A. T. , Baker, A. C. , & Dalzell, B. (2019). Phosphorus transport in intensively managed watersheds. Water Resources Research, 55, 9148–9172.

[jeq270339-bib-0013] Doydora, S. , Gatiboni, L. , Grieger, K. , Hesterberg, D. , Jones, J. L. , McLamore, E. S. , Peters, R. , Sozzani, R. , Van den Broeck, L. , & Duckworth, O. W. (2020). Accessing legacy phosphorus in soils. Soil Systems, 4, 74. 10.3390/soilsystems4040074

[jeq270339-bib-0014] Fausey, N. R. , Doering, E. J. , & Palmer, M. L. (1987). Purposes and benefits of drainage. In G. A. Pavelis (Ed.), Farm drainage in the United States: History, status and prospects. (pp. 48–51). USDA Economic Research Service.

[jeq270339-bib-0015] Gentry, L. , David, M. , Royer, T. , Mitchell, C. , & Starks, K. (2007). Phosphorus transport pathways to streams in tile‐drained agricultural watersheds. Journal of Environmental Quality, 36, 408–415. 10.2134/jeq2006.0098 17255628

[jeq270339-bib-0016] Gentry, L. E. , David, M. B. , Smith‐Starks, K. M. , & Kovacic, D. A. (2000). Nitrogen fertilizer and herbicide transport from tile drained fields. Journal of Environmental Quality, 29, 232–240.

[jeq270339-bib-0017] Guo, T. , Johnson, L. T. , LaBarge, G. A. , Penn, C. J. , Stumpf, R. P. , Baker, D. B. , & Shao, G. (2020). Less agricultural phosphorus applied in 2019 led to less dissolved phosphorus transported to Lake Erie. Environmental Science & Technology, 55, 283–291. 10.1021/acs.est.0c03495 33283499

[jeq270339-bib-0018] Hanrahan, B. R. , King, K. W. , Duncan, E. W. , & Shedekar, V. S. (2021). Cover crops differentially influenced nitrogen and phosphorus loss in tile drainage and surface runoff from agricultural fields in Ohio, USA. Journal of Environmental Management, 293, 112910. 10.1016/j.jenvman.2021.112910 34098350

[jeq270339-bib-0019] Jaynes, D. B. , Colvin, T. S. , Karlen, D. L. , Cambardella, C. A. , & Meek, D. W. (2001). Nitrate loss in subsurface drainage as affected by nitrogen fertilizer rate. Journal of Environmental Quality, 30, 1305–1314. 10.2134/jeq2001.3041305x 11476509

[jeq270339-bib-0020] Kamarath, B. , & Yuan, Y. (2023). Effectiveness of nutrient management for reducing phosphorus losses from agricultural areas. Transactions of the ASABE, 1, 77–88. 10.13031/jnrae.15572 PMC11238602 38993208

[jeq270339-bib-0021] King, K. W. , Williams, M. R. , Macrae, M. L. , Fausey, N. R. , Frankenberger, J. , Smith, D. R. , Kleinman, P. J. A. , & Brown, L. C. (2015). Phosphorus transport in agricultural subsurface drainage: A review. Journal of Environmental Quality, 44, 467–485. 10.2134/jeq2014.04.0163 26023966

[jeq270339-bib-0022] Kladivko, E. J. , Frankenberger, J. R. , Jaynes, D. B. , Meek, D. W. , Jenkinson, B. J. , & Fausey, N. R. (2004). Nitrate leaching to subsurface drains as affected by drain spacing and changes in crop production system. Journal of Environmental Quality, 33, 1803–1813. 10.2134/jeq2004.1803 15356241

[jeq270339-bib-0023] Ortas, I. , & Islam, K. R. (2018). Phosphorus fertilization impacts on corn yield and soil fertility. Communications in Soil Science and Plant Analysis, 49, 1684–1694.

[jeq270339-bib-0024] Osterholz, W. , Simpson, Z. , Williams, M. , Shedekar, V. , Penn, C. , & King, K. (2024). New phosphorus losses via tile drainage depend on fertilizer form, placement, and timing. Journal of Environmental Quality, 53, 241–252. 10.1002/jeq2.20549 38409568

[jeq270339-bib-0025] Patoine, M. , Hébert, S. , & D'Auteuil‐Potvin, F. (2012). Water quality trends in the last decade for ten watersheds dominated by diffuse pollution in Québec (Canada). Water Science and Technology, 65, 1095–1101. 10.2166/wst.2012.850 22378008

[jeq270339-bib-0026] Pease, L. A. , King, K. W. , Williams, M. R. , LaBarge, G. A. , Duncan, E. W. , & Fausey, N. R. (2018). Phosphorus export from artificially drained fields across the Eastern Corn Belt. Journal of Great Lakes Research, 44, 43–53.

[jeq270339-bib-0027] Pitcock, R. J. , & Jacinthe, P. A. (2025). Seasonal variation of enzymatically hydrolyzable organic phosphorus in agricultural drainage waters. Journal of Geophysical Research: Biogeosciences, 130, e2024JG008358.

[jeq270339-bib-0028] Schimel, J. , Balser, T. C. , & Wallenstein, M. (2007). Microbial stress‐response physiology and its implications for ecosystem function. Ecology, 88, 1386–1394. 10.1890/06-0219 17601131

[jeq270339-bib-0029] Sharpley, A. (2016). Managing agricultural phosphorus to minimize water quality impacts. Scientia Agricultura, 73, 1–8.

[jeq270339-bib-0030] Sheahan, S. G. (2025). Phosphorus loss in adjacent agricultural watersheds under different phosphorus fertilizer management [MS thesis, Indiana University]. 10.7912/CY7Y-X221

[jeq270339-bib-0031] Smith, A. S. , & Jacinthe, P. A. (2014). A mesocosm study of the effects of wet–dry cycles on nutrient release from constructed wetlands in agricultural landscapes. Environmental Sciences: Processes and Impacts, 16, 106–115. 10.1039/c3em00465a 24270400

[jeq270339-bib-0032] Smith, D. R. , Huang, C. , & Haney, R. L. (2017). Phosphorus fertilization, soil stratification, and potential water quality impacts. Journal of Soil and Water Conservation, 72, 417–424.

[jeq270339-bib-0036] Tarkalson, D. D. , & Mikkelsen, R. L. (2004). Runoff phosphorus losses as related to phosphorus source, application method, and application rate on a piedmont soil. Journal of Environmental Quality, 33, 1424–1430. 10.2134/jeq2004.1424 15254125

[jeq270339-bib-0033] Trentman, M. T. , Tank, J. L. , Royer, T. V. , Speir, S. L. , Mahl, U. H. , & Sethna, L. R. (2020). Cover crops and precipitation influence soluble reactive phosphorus losses via tile drain discharge in an agricultural watershed. Hydrological Processes, 34, 4446–4458.

[jeq270339-bib-0034] Venterink, H. O. , Davidsson, T. E. , Kiehl, K. , & Leonardson, L. (2002). Impact of drying and re‐wetting on N, P and K dynamics in a wetland soil. Plant and Soil, 243, 119–130.

[jeq270339-bib-0035] Withers, P. J. A. , Rodrigues, M. , Soltangheisi, A. , de Carvalho, T. S. , Guilherme, L. R. G. , Benites, V. D. E. M. , Gatiboni, L. C. , de Sousa, D. M. G. , Nunes, R. D. E. S. , Rosolem, C. A. , Andreote, F. D. , Oliveira, A. D. , Coutinho, E. L. M. , & Pavinato, P. S. (2018). Transitions to sustainable management of phosphorus in Brazilian agriculture. Scientific Reports, 8, 2537. 10.1038/s41598-018-20887-z PMC5803245 29416090

